# Effects of Immersive Virtual Reality with Treadmill in Subjects with Rett Syndrome: A Pilot Study

**DOI:** 10.3390/children11091110

**Published:** 2024-09-11

**Authors:** Daniele Panzeri, Michela Perina, Emilia Biffi, Martina Semino, Eleonora Diella, Tindara Caprì

**Affiliations:** 1 Scientific Institute IRCCS Eugenio Medea, Via Don Luigi Monza, 20, Bosisio Parini, 23842 Lecco, Italy; daniele.panzeri@lanostrafamiglia.it (D.P.); eleonora.diella@lanostrafamiglia.it (E.D.); 2 CARI (Airett Center Innovation and Research), Vicolo Volto S. Luca, 16, 37100 Verona, Italy; michela.perina@airett.it (M.P.); martina.semino@airett.it (M.S.); 3 Department of Life and Health Sciences, and Health Professions, Link Campus University, Via del Casale di S. Pio V, 44, 00165 Rome, Italy; tindara.capri@unime.it

**Keywords:** Rett, virtual reality, rehabilitation, treadmill

## Abstract

Background/Objectives: Rett syndrome is a rare neurodevelopmental disorder that can severely affect motor functioning, particularly walking. Previous training programs proposed treadmills as tools to increase walking endurance of patients with Rett syndrome, but these trainings did not include virtual reality (VR). The aim of this study was to assess the feasibility of a short treadmill training coupled to VR in girls with Rett syndrome. Methods: Nine patients with Rett syndrome underwent a 3-day treadmill walking program performed in semi-immersive VR. During the training, the happiness index and performance metrics were collected. At the end of the training parents filled out the Suitability Evaluation Questionnaire (SEQ) and, when feasible, patients underwent a gait assessment. Results: All the subjects recruited performed the three GRAIL sessions and parents showed a good satisfaction and considered the integration of treadmill and VR a good possibility for future rehabilitative programs. Participants showed greater satisfaction in environments requiring walking and their attention increased during training sessions, hypothesizing the feasibility of longer trainings with treadmill and VR. Data collected from gait analysis provided insights, although preliminary, concerning differences in gait pattern amongst the recruited subjects. Conclusions: Despite the small sample size and limited training duration, the paper suggests that a walking training with a treadmill combined with VR can represent a new strategy for Rett rehabilitation.

## 1. Introduction

Rett syndrome (RTT) is a rare severe neurodevelopmental disorder caused by mutations in the *MecP2* gene, which mostly affects females. Manifestation occurs generally in the first years of life, between 6 and 18 months of age with a regression of acquired skills, after an apparently normal or slightly delayed development. This regression is followed by a stable period, during which subjects with RTT show heterogeneous clinical situations [1]. The clinical representations are characterized by different degrees of cognitive disability and motor impairments such as fluctuation of muscle tone, loss of trunk and balance control, scoliosis and loss of purposeful hand use [2]. The later stage of the disease is characterized by osteotendineous retractions and progressive immobility. 

Considering walking abilities, Young et al. [3] observed that freezing, veering, foot dragging, parkinsonian shuffling, antepulsion or retropulsion and hand stereotypes characterize overground and treadmill walking of subjects with RTT. Isaias et al. [4] also underlined that subjects with RTT show a medium-lateral displacement during gait initiation, with a low activation of dorsiflexor of the ankle, a wide base of support and compensatory rotational movements of the trunk. The scarce representation of movement and the lack of feedforward control may be associated with sensory integration alterations, that may impact the walking function [5,6,7].

Lifelong motor rehabilitation is fundamental in subjects with RTT to maintain and improve their abilities and their quality of life through all the phases of the syndrome. According to a systematic review [8], the interventions performed for girls with RTT include education, traditional physiotherapy, environmental enrichment, hydrotherapy, treadmill training, sensory-based treatment, computerized systems and music therapy. Specific balance exercises, eye/feet coordination, walking on different surfaces and crossing obstacles are some of the goals in motor rehabilitation [4]. Regarding gait training, there is some evidence that walking on a treadmill may improve physical and aerobic fitness in subjects with RTT [9,10,11]. In these studies, kinematic data were not reported. 

Since the advent of virtual reality (VR), it has been extensively applied in the field of neurorehabilitation. The rationale of using VR is that it encourages and motivates the subjects to participate in the rehabilitation processes, while training in a safe environment which allows repeated learning trials [12]. When coupled with treadmill training, VR increased engagement and offered augmented feedback, improving motor function, movement kinematics, quality of life and participation in children with cerebral palsy and adults with chronic stroke [13,14,15]. 

Although results are promising, few studies investigated the effect of VR in subjects with RTT: Mraz et al. [16] reported, in their single case study, that training with a VR device seems to be effective in increasing hand use. In two other studies, subjects with RTT appear to be highly motivated by new technologies, particularly when including avatars in VR environments [17,18]. 

Considering the feasibility to use a treadmill with RTT and the engaging capabilities of VR, in this study we decided to assess if a short treadmill training coupled with VR activity is feasible in subjects with RTT. Specifically, the main aim of the present study was to investigate (1) the suitability of the system and the motivation of the patients during the training and (2) the motor responses of subjects with RTT in a setting of immersive virtual reality treadmill walking. 

## 2. Materials and Methods

### 2.1. Participants

Subjects affected by RTT were recruited through the Italian Association of Rett Syndrome (AIRETT). Inclusion criteria were diagnosis of RTT and ability to walk autonomously or with a support (Rett Syndrome Gross Motor Scale, Item 8 from 1 to 3 points). Exclusion criteria were inability to walk and deep cognitive disability with absence of cause–effect understanding, according to therapists with expertise on Rett syndrome.

The protocol was approved by the ethics committee of Scientific Institute Medea (protocol N. 72/21-CE approved on the 3 August 2021) and conducted in accordance with the Declaration of Helsinki. Participants’ parents or caregivers provided written informed consent. The trial was registered on clinicaltrials.gov (NCT05691582).

### 2.2. GRAIL System

The GRAIL is a system developed for gait analysis and rehabilitation in a semi-immersive VR environment. It is composed of a treadmill with two belts that can be driven at different speeds (0.1 to 3.6 Km/h) and equipped with two force platforms. The system integrates a motion platform that allows movements of antero-posterior pitch and lateral sway. To guarantee the patient’s safety, it is also equipped with two handrails and a harness for body weight support. The treadmill is surrounded by a 180° screen, on which it is possible to project different scenarios and visual feedback. The GRAIL includes a Vicon motion capture system (10 optoelectronic cameras, sample frequency 100 Hz) and 3 video cameras to perform motion detection and gait analysis, and this allows the integration of multi-sensorial feedback (visual, proprioceptive and auditory) during the training.

### 2.3. Study Design

Participants underwent an initial assessment of the cognitive and motor aspects using the Modified Adjusted Raven’s Colored Progressive Matrices, the Rett Assessment Rating Scale (RARS) and the Rett Syndrome Gross Motor Scale (RSGMS). These baseline assessments were performed by an experienced therapist prior to the access to the GRAIL.

The project consisted of 4 sessions of GRAIL activities in 1 week. During the first 3 sessions, exergames and augmented feedback activities were proposed. During the fourth session a gait analysis was conducted for the subjects with a suitable motor and behavioral level.

In every session, before the beginning of GRAIL activities, an adaptation time of five minutes was proposed in order to allow an acclimatization to the GRAIL environment. Moreover, a further adaptation time was conceded anytime the exercise changed, according to the need of each subject.

During all the sessions, a video recording was conducted to detect facial expression of the subjects, in order to use these data to compute the “happiness index”. The exergames were administered until the therapists detected any sign of discomfort by the subject.

The exercises to be used during the sessions were defined by two therapists that have experience with GRAIL training and two therapists with expertise on Rett syndrome. The protocol included 4 exergames that were administered every session to all the subjects. The order of execution of the activities changed at every session.

**Figure 1:**
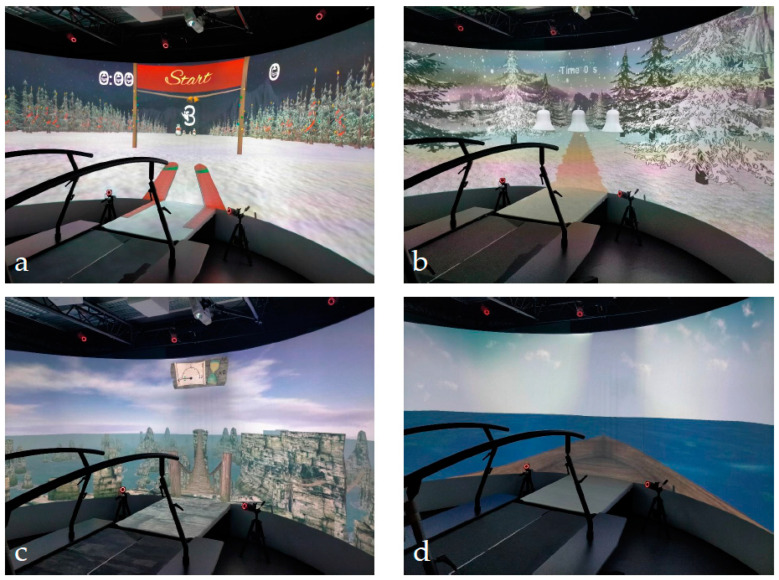
GRAIL environments. (**a**) APP1, interaction with virtual reality; (**b**) APP2, walking; (**c**) APP3, dynamic Balance; (**d**) APP4, static balance.

The chosen exergames had different aims, as follows:

APP1—INTERACTION WITH VIRTUAL REALITY: this application aims to verify the ability to deal with the visual feedback and to use it to interact with the exergame. This game (licensed by Motek with the name “MM Christmas 2014”) is a simulation of a slalom between snowmen. Subjects wear virtual skis and must move their center of mass (detected by passive reflective markers located on the pelvis) on the platform to interact with the environment. The treadmill is stationary. Particularly, they have to move their pelvis forward to increase the speed or backward to reduce it, while they have to shift their pelvis right or left to let the skis slide correspondingly. From the therapist console it is possible to set different task difficulties, modifying the distance between each snowman or adapting the sensitivity to the movement of the patient in order to increase or reduce the speed (Figure 1a).

APP2—WALKING: this application (licensed By Motek with the name “MM Christmas 2012”) was chosen to promote engagement during linear walking. In this exergame, subjects are immersed in a snowy wood, with joyful background music. During this game it is possible to set different speeds, according to participant abilities (Figure 1b). 

APP3—DYNAMIC BALANCE: this application aims to stimulate the capacity to modulate gait characteristics of participants in relation to the slope they encounter. In this application (licensed by Motek with the name “rope bridge”) the subjects walk over a rope bridge. According to the capacity shown by the patient, the therapist can set different walking speeds and different degrees of slope. Moreover, it is possible to deliver lateral sway perturbation, simulating blowing wind, in order to train the ability of the subject to react to external stimulations (Figure 1c).

APP4—STATIC BALANCE: this application, developed at IRCCS Medea, aims to stimulate the balance reaction of the subject to external perturbations. The game presents a marine environment in which the subject must stand over a boat (projected on the GRAIL platform) rolling over the waves of the sea. During this exercise, the platform pitches forward and backward, with a movement amplitude related to the height and speed of the waves, as set by the therapist (Figure 1d).

Exercise parameters were customized regarding the patients’ engagement and need, modifying speed, inclination and difficulty of the exercise. 

#### 2.3.1. Baseline Evaluation

At baseline, the following data were collected.

*Rett Syndrome Gross Motor Scale (RSGMS)*

This scale aims to assess gross motor abilities in individuals with RTT. It has 15 items, subdivided into three subscales (sitting subscale, standing and walking subscale and challenge subscale) and includes abilities such as sitting, standing, transitions, walking and going up and down the stairs. The score is assigned according to needed assistance to complete every action, from 0 (meaning no assistance) to 4 (meaning maximum assistance or not being able to complete the action). RSGMS has demonstrated strong internal consistency both for the total score and for each subscale (Cronbach’s alpha coefficient for total score = 0.96, for sitting subscale = 0.83, for standing and walking subscale = 0.97, for challenge subscale = 0.85) and a high repeatability (correlation coefficient = 0.99, 95% CI 0.93–0.98) [19].

*Modified Adjusted Raven’s Colored Progressive Matrices*

A modified version of the Adjusted Raven’s Colored Progressive Matrices was used in this study to assess general cognitive abilities of the participants. Only Series A was administered to the subjects, and the items were placed separately in front to the subjects. Every item was presented three times to every subject, randomizing position of the target. The subsequent table was presented after two correct consecutive answers. The test was interrupted if the subject gave three wrong answers. The score of the test was achieved by summing the number of right answers for each item [20].

*Rett Assessment Rating Scale*

The Rett Assessment Rating Scale (RARS) is an RTT-specific scale that aims to evaluate the severity of the syndrome. It includes 31 items, which cover many aspects of the clinical features, such as behavioral, emotional, motor and communication aspects, and investigates in depth the presence and intensity of the main characteristics of RTT, such as anxiety, epilepsy, breathing dysregulation, bruxism and muscular tone. Every item can be rated from 1 (within normal limits) to 4 (strong abnormality). It showed high internal consistency for both total and subscale rates (Cronbach’s alpha coefficient for total score = 0.91, for the subscales = 0.81–0.93) [21,22].

#### 2.3.2. Outcome Measures

The following outcome measures were collected during the three sessions of training (hereinafter, T1, T2 and T3): happiness index, endurance time, performance speed and attention focus on scenario. At the end of the training, parents were asked to fill in a modified version of the Suitability Evaluation Questionnaire (SEQ) and the patients underwent a 3-dimensional gait analysis if possible.

*Happiness Index*

This parameter is derived from Van der Maat’s taxonomy, which contains an analysis of the behavior of people with severe intellectual disabilities. Only seven behaviors out of the twelve main categories of the taxonomy of Van der Maat were taken into account. The behaviors were: gaze direction, sounds, mouth movements, physiological reactions and hand gestures. They were recorded by a camera in front of the subjects. Scores were assigned as 0 if the parameter (behavior) was not present in the record and as 1 if it was present. The overall score of the happiness index was defined as the sum of the scores [23,24,25]. This assessment was performed during every session (i.e., at T1, T2 and T3).

*Endurance Time*

During all the sessions, the time of execution of each exergame was registered. The activity was stopped when the participant showed discomfort or excessive inquietude, according to the experimenters.

*Performance Speed*

In APP2 and APP3, during which subjects had to walk over the treadmill, we recorded the maximum speed it was possible to reach. Performance speed was measured at T1, T2 and T3.

*Attention Focus on Scenario*

The number of seconds of attention on each scenario was measured through the video recording analysis. The attention span started when the subject looked at the object of focus requested by each APP and continued until the subject looked away from the object and stared into space, as previously performed [17]. The type of requested action for each APP is described in the study design section.

*Suitability Evaluation Questionnaire (SEQ)*

At the end of the training, parents were asked to complete a modified version of the Suitability Evaluation Questionnaire (SEQ). Particularly, we modified the SEQ developed by Gil-Gómez et al. [26] to allow the parents to assess the suitability of the GRAIL system for the subjects. SEQ was indeed developed by Gil-Gómez and collaborators to assess usability, acceptance and security of use of VR environments/devices. It includes 13 questions rated as a 5-point Likert scale and 1 open question. The SEQ assesses enjoyment, sense of being in the system, feeling of success and control, realism, easy-to-understand instructions and general discomfort of the system. Furthermore, the SEQ measures specific symptoms related to cybersickness such as nausea, eye discomfort and disorientation. Finally, one question assesses the perceived usefulness of the GRAIL system for patient rehabilitation. The total SEQ score ranges between 13 and 65 (excellent suitability).

*Gait Analysis*

On the last day of training, a gait analysis was conducted when it was considered feasible by experimenters. The criterion to access to this assessment was that the subject had to show a minimum of 5 min of autonomous walking. Subjects could use their hands to hold or reach the support, but no physical interactions with other persons was allowed during this period. 

To accomplish the analysis, 26 passive reflective markers were located over the patient according to the model proposed by Vicon (HBM2 trunk and lower limb) for the analysis of lower limb and trunk movements. For their safety, all the subjects had to wear the harness provided by the GRAIL manufacturer. The recordings were executed after a period of adaptation to the movement of the treadmill of at least 3 min. An adaptation time of 10 min is considered to be more reliable, but considering the reduced attention and walking resistance shown by these patients, it was decided to require a shorter adaptation period. For every subject, at least 4 blocks of gait acquisition were collected, each consisting of a minimum of 20 steps. In this study, the final gait acquisition was considered for further data analysis since it was evaluated as the most reliable.

Gait data were extracted by using the Gait Offline Analysis Tool (GOAT by Motek). GOAT synchronizes 3D motion capture, force plate data and video recordings that are displayed, allowing identification of reliable steps. GOAT filters GRAIL data with a low-pass 2nd order Butterworth filter, with a cut-off frequency equal to 6 Hz, and performs gait event detection and standardized step selection. A visual inspection was then performed to eliminate incorrect steps. Specifically, a step was eliminated if the foot is located both on the left and the right belt of the treadmill, preventing a correct detection of forces. Finally, GOAT normalizes the steps on 100 samples, returning a single value for the space–time parameters and time series for kinetic and kinematic parameters.

### 2.4. Statistic Analysis

Data were analyzed using SPSS version 24.0 for Windows. The descriptive statistics of the dependent variables were computed as median and interquartile range (IQR). Considering the small sample size, a non-parametric analysis was performed. Accordingly, the Friedman test was used to test if scores of parameters (happiness index, endurance time, performance speed and attention focus on scenario) were significantly different among the three VR environments (APP2, APP3 and APP4) and among the three sessions (T1, T2 and T3). Alpha level was set equal to 0.05. If the Friedman test highlighted statistically significant differences, a post hoc analysis with the Wilcoxon paired test with Bonferroni correction was used (considering *p*-values < 0.016 as significant). 

## 3. Results

Nine female subjects (age range 6–44 years old, median (IQR) = 16 (4)) were recruited according to the inclusion criteria. Data are reported in Table 1. 

As shown in Table 1, the patients had a level of severity of syndrome from severe to mild (RARS total scores), with a median (IQR) value of 60.5 (8.5), meaning that they showed severe physical, language and social impairments. This clinical feature was expected because RTT is a severe disorder associated with deficits in several domains. With regard to motor functioning, it was moderately associated with the degree of severity of RTT (according to the RSGMS total scores).

All participants performed the three training sessions (T1, T2 and T3) in one week according to the protocol. 

After the first training, it was decided to remove APP1 from the list of the exergames. This app was selected in order to assess the ability of subjects to understand the motor request and to interact with the system by moving their body left or right to perform the exergame. Unluckily, this task was too difficult for the subjects and, when this game was not conducted properly, the visual biofeedback was too confusing. 

All parents completed the SEQ. Gait analysis was possible only for four subjects. The following sections show results related to outcome measures, i.e., happiness index, endurance time, performance speed, attention focus on scenario (Table 2), SEQ and gait analysis. 

### 3.1. Happiness Index 

The happiness index scores significantly differed in the three environments (*p* = 0.013, Friedman test). Particularly, there were statistically significant differences in the level of happiness index between APP3 (dynamic balance) and APP4 (static balance) (*p* = 0.014) with participants showing more happiness-promoting behaviors in the bridge scenario proposed in APP3. In general, the snow scenario of APP4 was the one with lower values of the happiness index.

With reference to happiness index scores observed during T1, T2 and T3 for each scenario, there was only a trend in the waves scenario proposed in APP4 (*p* = 0.066, Friedman test). This means that only in APP4 is it possible to observe a positive trend of happiness-promoting behavior among the three sessions.

### 3.2. Endurance Time

The Friedman test did not show statistically significant differences among environments in terms of endurance. However, there is a trend in data suggesting that participants performed the scenario of APP4 for a longer time, before showing discomfort signs, with respect to the other environments.

There were no differences among T1, T2 and T3 for all the three applications.

### 3.3. Performance Speed

Considering the performance speed for APP2 and APP3, no significant difference was found. This means that participants walked on the treadmill in these two scenarios using similar speed. Considering the sessions, the Friedman test highlighted a statistically significant difference for APP3 (*p* = 0.009). The comparison between time points with the Wilcoxon test resulted in *p* = 0.042 (T1 vs. T2), *p* = 0.027 (T1 vs. T3) and *p* = 0.498 (T2 vs. T3) suggesting that performance speed increased after T1, although *p*-values were not statistically significant considering the Bonferroni correction (*p* < 0.016).

### 3.4. Attention Focus

With reference to the attention focus on each scenario, no significant difference was found among them. This means that participants looked at each scenario for similar numbers of seconds. 

With reference to attention focus scores observed at T1, T2 and T3 for each scenario, there were statistically significant differences for APP3 (*p* = 0.005, Friedman test) and APP4 (*p* = 0.013, Friedman test) among sessions. Regarding APP3, T1 differed from T3 (*p* = 0.021, Wilcoxon test). Considering APP4, differences between T1 vs. T3 and T2 vs. T3 (Wilcoxon: *p* = 0.021 and *p* = 0.028, respectively) were observed. Although *p*-values were not statistically significant according to Bonferroni correction, these results suggest that attention focus improved over time.

### 3.5. SEQ

Parents reported a median SEQ score of 56 (IQR = 13) (range 44–62) (median normalized suitability score of 0.82 (IQR = 0.2)), highlighting a good suitability of the GRAIL system according to parents’ opinion. Particularly, patients enjoyed the system (Q1, median (IQR) = 5 (2)), they did not have nausea or dizziness (Q8, median (IQR) = 1 (0)) and were not confused or disoriented (Q10, median (IQR) = 1 (1)). Parents thought that GRAIL could be helpful for their daughters’ rehabilitation (Q11, median (IQR) = 4 (2)).

### 3.6. Gait Analysis

**Figure 2:**
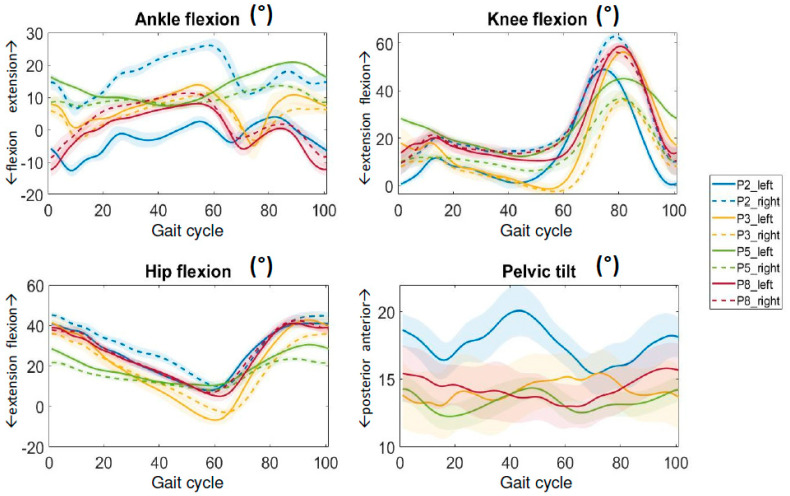
Gait analysis during flat walking over the GRAIL. Ankle, knee, hip and pelvis kinematics in the sagittal plane are reported. Blue lines refer to P2, yellow lines to P3, green ones to P5 and red ones to P8. Continuous lines refer to the left side while the dashed ones to the right side. The lines show the mean values of the steps collected while the colored shadows represent the standard deviation of the curves.

Considering the four patients that were able to perform the gait analysis, they showed different gait patterns, as shown by kinematics graphs (Figure 2) and spatio-temporal and kinematic data reported in Table 3, but with some common aspects: as a whole, the group analyzed seems to show an increased stiffness, a marked reduction in the ankle range of motion (ROM) and a lack of plantar flexion during the push-off phase.

P2 was the subject who could reach the fastest speed amongst the analyzed group. In fact, P2 could walk at a mean speed of 0.66 m/s, with a mean stride length of 0.87 m. We measured 33 steps. P2’s gait pattern was characterized by a marked asymmetry between the left and right lower limbs, as reported in Table 3. P2 used the right side more as a support and the left side for advancing, as evidenced by the comparison between the 79% of stance phase on the right side and 66% on the left. Correspondingly, from the kinematics graphs detailed in Figure 2, it is possible to notice that the right side was more flexed, both at the hip and the knee, and the ankle more dorsiflexed during the whole gait cycle. In contrast, the left side remained more extended, and it was characterized by stiffness of the knee during the swing phase and ankle plantarflexion throughout the cycle of gait. This is further confirmed by the difference in the ROM data between left and right lower limbs.

P3 walked with a mean speed of 0.5 m/s and a mean length of the stride of 0.77 m. We measured 33 steps. Kinematics data show a reduction in the ROM of all the sections of the two lower limbs (consistent with a global stiffness), but it was more evident on the right side. Among the analyzed subjects, P3 was able to produce the greatest extension both in hip and knee as observable in the graph in Figure 2.

P5 could walk only with a very low speed, equal to 0.25 m/s. We measured 57 steps. P5’s mean stride length was only 0.20 m. The gait pattern was characterized by parkinsonian shuffling and severe stiffness, with reduction of the active ROM in all lower limb sections and, as a consequence, very small and fast steps. Data from kinetics report the same reduction of any activity of push over the ground.

P8 walked with a mean speed of 0.5 m/s and a mean stride length of 0.68 m. We measured 39 steps. Differently from the other subjects, kinematics data from the lower limbs of this subject were more similar to kinematics of healthy subjects. The hips appear to be slightly more flexed and showed an external rotation during the stance phase. From kinetics a reduction during distal push-off is evident.

## 4. Discussion

In the literature, several studies have been carried out to describe the feasibility and efficacy of a walking training using a treadmill in populations of subjects affected by RTT [9,10,11]. Layne et al. [9] analyzed the gait pattern of 17 girls with RTT walking on a treadmill with a gradual increase in speed (0.1 m/s every 20 s). The authors underlined that participants could rely on sensory feedback to adapt their gait to changes in speed of the treadmill. Similarly, Larsson et al. [11] demonstrated that, while walking on a treadmill, subjects with RTT can adapt their own speed until their maximum speed. These results seem to suggest the possibility to train subjects with RTT with a treadmill in order to increase their walking speed. Lotan et al. [10] proposed a low-intensity daily program with a treadmill for 2 months for four subjects with RTT. The intervention improved physical and aerobic fitness of the participants suggesting that a treadmill can train the cardio-pulmonary functioning in this population. In these two studies, the authors measured physiologic signals, but not kinematics data.

On the other hand, several studies have been conducted to verify the interaction of RTT subjects in a VR environment [16,17,18], but they were mainly addressed to upper limb functions and cognitive interaction. 

Therefore, this is the first study in which a short training combining treadmill and VR environment is proposed for subjects with RTT. The study investigated (1) the suitability of the system and the motivation of the patients during the training and (2) the motor responses of subjects with RTT during training. The first aim was fulfilled considering the adherence of patients to the training, the SEQ, the happiness index and the attention focus to scenarios. The second aim was achieved by means of the endurance time and performance speed measures as well as gait analysis data when available. 

Considering the feasibility, all the subjects recruited were able to complete the three GRAIL sessions and with four of them it was possible to conduct a gait analysis. Moreover, the parents of participants showed a good satisfaction and considered the integration of the treadmill and VR a good possibility for future rehabilitative projects.

Considering the happiness index, the participants showed greater satisfaction in APP2 and APP3 during which they walked over the treadmill, in comparison to APP4, during which the task was performed in static conditions. 

Interestingly, our preliminary data may suggest that patients with RTT increase their attention toward the scenario and task during training sessions, hypothesizing the feasibility of longer trainings with a treadmill and VR.

Considering the second aim of the paper, according to data collected, the most durable activity was APP4. This may be due to the fact that it was the only activity that did not require walking and it is possible that patients got tired more slowly.

Regarding gait speed, data reported in this manuscript may suggest that the gait speed can gradually increase during the training.

The gait analysis was feasible in four patients. The median walking speed was 0.5 ms^−2^, a value higher than the mean speed reached during the three sessions. This is due to the fact that only patients that showed greater endurance and safety during walking could accede to gait analysis, while data for mean speed during training were collected from all the subjects recruited. With this median speed, participants had a median stride time equal to 1.33 s that is longer than the one reported by Layne et al. [9], where the median stride time at a similar speed was between 1.21 s and 1.17 s.

The four subjects that underwent the gait assessment showed some common characteristics, such as a global stiffness and, accordingly, the active ROM reduction in lower limbs, the lack in push-off activity in the pre-swing phase and an increase in the stance time. 

Considering the ROM, the study of Layne et al. included participants with severe stiffness that limited hip movement to a maximum ROM of 12° and knee ROM to a maximum of 10° [9]. In contrast, the participants that underwent the gait analysis in this study had a median hip ROM of 36.25° and a median knee ROM of 49.25°. This means that the subjects that could undergo the gait analysis showed a less impaired gait pattern.

The lack of plantar flexion during the pre-swing phase (healthy subjects usually exhibit 15° of plantar flexion [27]) seems to suggest the deficiency in push-off activity of gastrocnemius and soleus muscles, and this can contribute to the reduction in stride length and in walking speed.

Finally, the increase in stance time, directly related to the duration of single-limb support, is a consequence of the difficulty in properly managing the balance of center of mass.

On the other hand, additionally to these common behaviors, each subject showed peculiar characteristics. The mean walking speed, for example, was very different between P2 (0.66 m/s) and P5 (0.25 m/s). P2 was markedly asymmetrical, while P8 showed similar kinematics for left and right lower limbs; P5 was the most symmetric, but presented the most limited ROM, and this can explain this symmetry. During walking, P3 could achieve hip and knee extension, particularly in the late stance, while P8 remained with the knee flexed during the whole gait cycle. These results suggest that in addition to some common characteristics, subjects with RTT show specific gait pattern alteration.

For a correct interpretation of these results, we have to consider the limitations of this study. This is indeed a pilot study with a small simple size. Therefore, it is necessary to increase the sample size to confirm the results obtained. In addition, the age range of the sample is broad (6–44 yo); nevertheless, this is common in research papers about subjects with RTT. However, our results must be verified in a larger sample. On the other hand, it is important to consider that RTT is a rare syndrome, limiting the possibility to recruit a big sample with a narrow age range. Future studies could use the methodologies of this pilot study with a large sample of patients, taking into account their age, to replicate our findings. 

Secondly, considering that for safety reasons the presence of any person in close proximity of the device was not possible, we decided to take a cautious approach (e.g., stopping the treadmill as soon as the subjects showed signs of discomfort or started veering). This limited the possibility to determine the effective maximum speed during the training.

Finally, although the purpose of this study was to verify the suitability of a treadmill training plus VR in patients with RTT and not its efficacy, we must consider the short duration of each session and the reduced dose of the therapy. Indeed, this study was preliminary to future research that will recommend more intensive training with this system as previously performed [14]. 

Nevertheless, this system may have strong clinical implications in the rehabilitation practice of children, adolescents and adults with RTT. Indeed, enhanced motivation and engagement fostered by exergames in virtual reality can support longer training sessions that may cause improved walking endurance. This may consequently boost autonomy in daily life activities, thus improving quality of life of patients with RTT. It is therefore possible to hypothesize that treadmill training combined with VR could be a valid treatment for motor functions in RTT. Future research will be conducted to validate this type of treatment in a randomized controlled trial.

## 5. Conclusions

In conclusion, this study demonstrated that subjects with RTT tolerated the treadmill plus VR training well, without showing signs of discomfort or dizziness. The endurance time in all the activities increased gradually. Among the activities proposed, subjects liked the three applications that required more automatic movements more, such as walking and maintaining balance over the platform. Furthermore, kinematic data during walking were reported for the first time in this population, highlighting some common features but also a high variability in this population.

Hence, the present study suggests that this approach, consisting of a treadmill integrated with VR, is feasible and tolerable in subjects with RTT.

## Figures and Tables

**Table 1:** Characteristics of participants.

Participants	Age	Level of Severity ^1^	RARS Total Scores ^2^	RSGMS Total Scores ^3^	Raven Total Scores ^4^
P1	11	Moderate	57	9	0
P2	17	Mild	52.5	37	1
P3	16	Moderate	54	32	3
P4	16	Moderate	60	29	2
P5	44	Mild	53.5	30	1
P6	14	Moderate	68	32	1
P7	18	Moderate	57.5	34	3
P8	35	Moderate	61	32	2
P9	6	Severe	84	35	1

^1^ Levels of severity according to the total scores of RARS (mild = 0–55; moderate = 56–81; severe = >81); ^2^ Rett Assessment Rating Scale (RARS); ^3^ Rett Syndrome Gross Motor Scale (RSGMS); ^4^ Modified Adjusted Raven’s Colored Progressive Matrices. P: participants.

**Table 2:** Median (M) and interquartile range (IQR) of happiness index, endurance time, performance speed and attention focus for each scenario in the three training sessions.

Parameters	Scenario	T1 M (IQR)	T2 M (IQR)	T3 M (IQR)
Happiness index ^1^	APP2	4 (2)	4 (2)	3 (2)
	APP3	3 (2)	3 (1)	3 (0)
	APP4	2 (1)	2 (1)	3 (1)
Endurance time (s) ^2^	APP2	180 (160)	280 (180)	240 (180)
	APP3	240 (230)	180 (80)	240 (90)
	APP4	300 (210)	300 (120)	300 (60)
Performance speed (m/s) ^3^	APP2	0.4 (0.1)	0.4 (0.1)	0.4 (0.1)
	APP3	0.3 (0.1)	0.4 (0.1)	0.4 (0.1)
Attention focus (s) ^4^	APP2	120 (125)	91 (118)	206 (198)
	APP3	54 (154)	160 (148)	195 (175)
	APP4	120 (93)	105 (92)	165 (90)

^1^ Presence or absence of seven behaviors related to Van der Maat’s taxonomy (min. 0–7 max.); ^2^ Time of execution of each exergame measured in seconds; ^3^ Meters per second; ^4^ Attention time on each scenario measured in seconds.

**Table 3:** Mean and standard deviation (M ± std) of spatio-temporal and kinematic parameters of each participant performing gait analysis over the GRAIL (P2, P3, P5 and P8) are reported. The last column reports the median values and interquartile ranges of spatio-temporal and kinematic parameters considering all participants and legs. R: right (leg); L: left (leg); ROM: range of motion; IQR: interquartile range; ° degrees.

Gait Parameter	Side	P2	P3	P5	P8	Median (IQR)
Walking speed		0.66 ± 0.03	0.50 ± 0.02	0.25 ± 0.02	0.50 ± 0.04	0.50 (0.10)
Stance phase %	R	79.59 ± 1.45	73.79 ± 1.43	71.20 ± 2.36	67.50 ± 1.99	70.77 (3.27)
	L	66.43 ± 1.24	71.92 ± 2.30	70.34 ± 1.98	69.65 ± 1.72	
Stride time (s)	R	1.31 ± 0.05	1.57 ± 0.06	0.88 ± 0.07	1.35 ± 0.07	1.33 (0.20)
	L	1.32 ± 0.05	1.57 ± 0.06	0.87 ± 0.06	1.34 ± 0.08	
Step length (m)	R	0.42 ± 0.05	0.39 ± 0.02	0.12 ± 0.02	0.36 ± 0.04	0.37 (0.13)
	L	0.44 ± 0.03	0.39 ± 0.04	0.10 ± 0.02	0.31 ± 0.03	
Ankle ROM °	R	20.22 ± 3.21	15.63 ± 1.65	7.15 ± 1.36	21.48 ± 2.19	18.85 (5.82)
	L	17.48 ± 2.16	20.82 ± 4.51	13.76 ± 1.06	21.54 ± 1.90	
Knee ROM °	R	55.87 ± 3.42	38.99 ± 4.00	31.38 ± 3.33	49.21 ± 4.10	49.24 (13.93)
	L	49.98 ± 3.26	58.59 ± 3.76	33.10 ± 2.12	49.28 ± 1.86	
Hip ROM °	R	36.64 ± 2.05	40.04 ± 1.87	16.24 ± 1.98	36.04 ± 2.46	36.25 (6.29)
	L	34.62 ± 2.66	51.54 ± 3.81	20.94 ± 1.82	36.46 ± 1.70	

## Data Availability

Data are openly available after paper acceptance in Zenodo at https://doi.org/10.5281/zenodo.10038485 (accessed on 1 June 2024).
